# *MAP3K1* and *MAP2K4* mutations are associated with sensitivity to MEK inhibitors in multiple cancer models

**DOI:** 10.1038/s41422-018-0044-4

**Published:** 2018-05-24

**Authors:** Zheng Xue, Daniel J. Vis, Alejandra Bruna, Tonci Sustic, Sake van Wageningen, Ankita Sati Batra, Oscar M. Rueda, Evert Bosdriesz, Carlos Caldas, Lodewyk F. A. Wessels, René Bernards

**Affiliations:** 1 Division of Molecular Carcinogenesis, Oncode Institute, The Netherlands Cancer Institute, Plesmanlaan 121, Amsterdam, 1066 CX The Netherlands; 2 Department of Oncology and Cancer Research UK Cambridge Institute, Li Ka Shing Centre, University of Cambridge, Cambridge, CB2 0RE UK; 3 Cambridge Breast Unit, NIHR Cambridge Biomedical Research Centre and Cambridge Experimental Cancer Medicine Centre, Cambridge University Hospitals NHS Foundation Trust, Cambridge, CB2 2QQ UK

## Abstract

Activation of the mitogen-activated protein kinase (MAPK) pathway is frequent in cancer. Drug development efforts have been focused on kinases in this pathway, most notably on RAF and MEK. We show here that MEK inhibition activates JNK-JUN signaling through suppression of DUSP4, leading to activation of HER Receptor Tyrosine Kinases. This stimulates the MAPK pathway in the presence of drug, thereby blunting the effect of MEK inhibition. Cancers that have lost *MAP3K1* or *MAP2K4* fail to activate JNK-JUN. Consequently, loss-of-function mutations in either *MAP3K1* or *MAP2K4* confer sensitivity to MEK inhibition by disabling JNK-JUN-mediated feedback loop upon MEK inhibition. In a panel of 168 Patient Derived Xenograft (PDX) tumors, *MAP3K1* and *MAP2K4* mutation status is a strong predictor of response to MEK inhibition. Our findings suggest that cancers having mutations in *MAP3K1* or *MAP2K4*, which are frequent in tumors of breast, prostate and colon, may respond to MEK inhibitors. Our findings also suggest that MAP3K1 and MAP2K4 are potential drug targets in combination with MEK inhibitors, in spite of the fact that they are encoded by tumor suppressor genes.

## Introduction

The genetic aberrations that lie at the heart of cancer can create a dependency, a situation referred to as “oncogene addiction.”^1^ Inhibition of these oncogenic signals using drugs that selectively inhibit these so called “driver” pathways often leads to massive clinical responses. It is estimated that over 30% of all human cancers are driven by mutations in *RAS* genes,^2^ but with the notable exception of *KRAS G12C* mutant RAS proteins, RAS proteins have mostly resisted drug development efforts.^3,4^ RAS proteins connect growth factor signaling to multiple downstream pathways, including the RAF-MEK-ERK pathway (also known as the mitogen activated protein kinase (MAPK) pathway) and the PI3K pathway. These pathways contribute to oncogenesis through stimulation of cell proliferation and escape from apoptosis. Given the mostly “undruggable” nature of RAS proteins, drug development efforts have been focused on the kinases in the pathways downstream of RAS. Indeed, inhibition of RAF-MEK-ERK kinases can result in decrease in tumor cell proliferation and induce apoptosis.^5,6^ Many pharmaceutical companies have developed MEK kinase inhibitors, but the clinical benefit of these inhibitors has been disappointing to date.^7–9^ A notable exception is the use of MEK inhibitors in *BRAF* or *NRAS* mutant melanomas.^10,11^ Thus, identifying predictive biomarkers for MEK inhibitor response and potential combination therapies that enhance MEK inhibitor effectiveness is essential for the future clinical use of these drugs.

Recent large-scale genomic studies have identified oncogenic driver mutations in multiple cancers, including recurrent mutations in *MAP3K1* and *MAP2K4*.^12–14^ The *MAP3K1* and *MAP2K4* mutations are loss-of-function mutations, including nonsense and frame shift mutations and a missense mutation (Ser56Leu), which interferes with MAP2K4 kinase activity.^12,13,15^ The highest mutation frequency in these genes is found in invasive ductal breast cancers: *MAP3K1* 9% and *MAP2K4* 7%,^16^ followed by cancers of prostate, stomach and diffuse large B cell lymphoma^16–21^ (http://www.cbioportal.org).

DUSP4, which dephosphorylates JNK to inhibit its kinase activity, mediates the crosstalk between MEK-ERK and JNK-JUN pathways. ERK is known to inhibit JNK via an induction of DUSP4 mRNA and protein expression, while inhibition of MEK-ERK signalling activates JNK-JUN signaling through inhibition of the DUSP4.^22,23^ The MAP3K1-MAP2K4-JNK cascade activates JUN, which in combination with FOS, forms the Activator Protein-1 (AP-1) transactivator complex that controls a number of cellular processes including differentiation, proliferation, and apoptosis.^24^ The significant number of *MAP3K1* and *MAP2K4* mutations in different types of cancers is still poorly understood due to their dual roles in cell survival and apoptosis. MAP3K1 can promote cell survival through activation of MAP2K4/7-JNK-JUN, MAP2K1/2-ERK1/2 and NF-κB, while a MAP3K1 kinase domain generated by caspase-3 cleavage can induce apoptosis.^17^ Consequently, both activating and inactivating mutations in these genes are seen in cancer^2^ (http://www.cbioportal.org). In addition, it is not clear whether mutations in *MAP3K1* or *MAP2K4* cause a vulnerability that can be targeted with specific drugs. We show here an unexpected relationship between loss-of-function mutations in *MAP3K1* and *MAP2K4* and response to MEK inhibitors.

## Results

### Recurrent MAP3K1 and MAP2K4 mutations sensitize cancer cells to MEK inhibitors

**Fig. 1:**
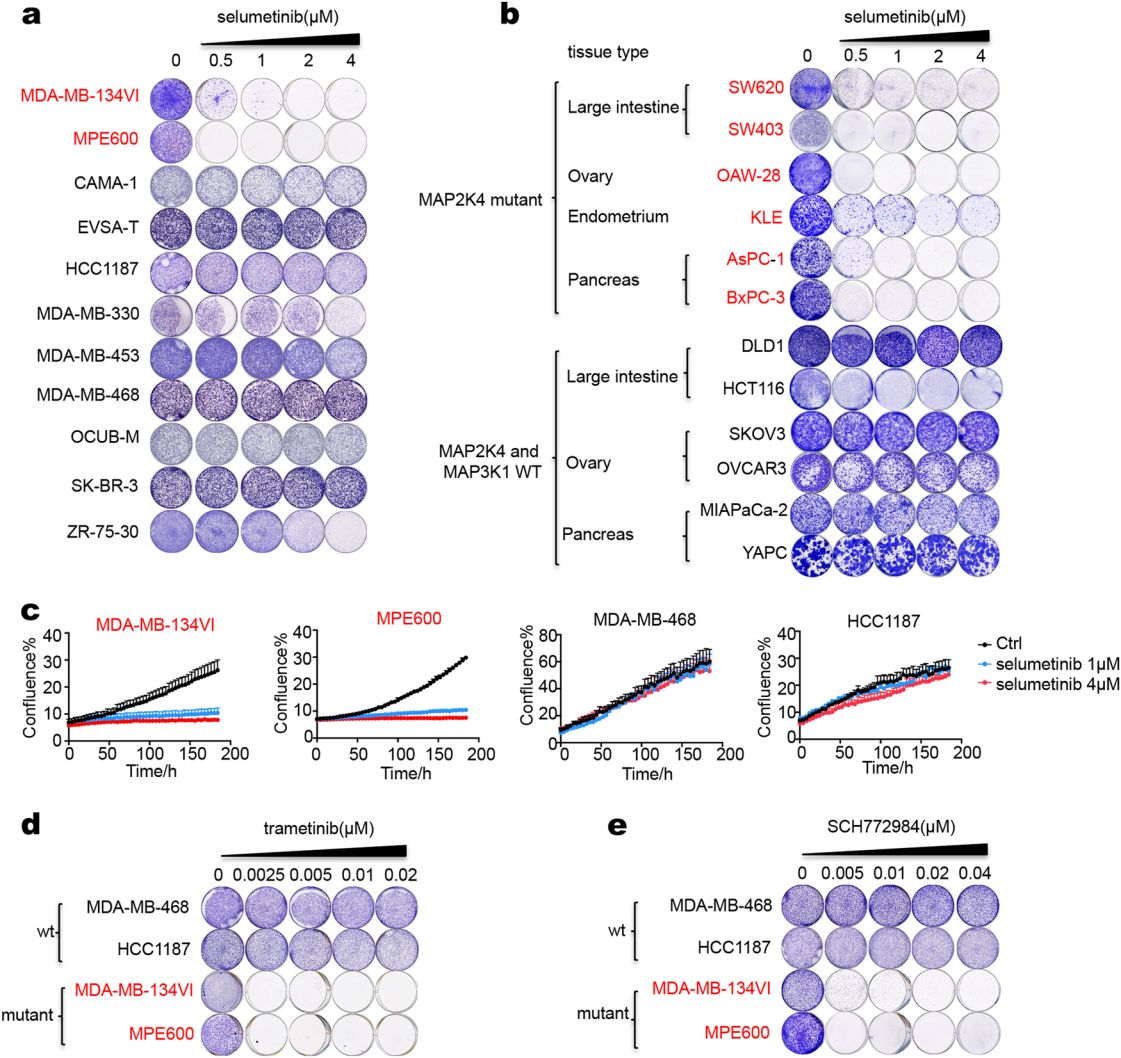
*MAP2K4* mutant cell lines respond to MEK inhibition. **a** A breast cancer cell line panel including two *MAP2K4* mutant breast cancer cell lines (red) and nine wild-type breast cancer cell lines (black) were cultured in medium containing the indicated concentration of selumetinib for two weeks. After this, cells were fixed and stained. **b** *MAP2K4* mutant (red) and wild-type (black) cell lines were cultured in medium containing the indicated concentration of selumetinib for two weeks. After this, cells were fixed and stained. **c** Cell proliferation curves of 4 breast cancer cell lines. Two *MAP2K4* mutant breast cancer cell lines (red) and two wild-type breast cancer cell lines (black) were cultured in medium containing the indicated concentration of selumetinib. Percent confluence over time was monitored using an IncuCyte real-time imager. **d**,**e** Four breast cancer cell lines of indicated MAP2K4 mutation status were cultured in medium containing the indicated concentration of trametinib (**c**) or SCH772984 (**d**) for 2 weeks. After this, cells were fixed and stained

To study whether the *MAP3K1* and *MAP2K4* mutations identified in breast cancers give rise to a vulnerability that can be exploited therapeutically, we used a panel of breast cancer cells lines that we sequenced previously.^12^ Among the 11 breast cancer cell lines, we found that MDA-MB-134VI and MPE600 had inactivating mutations in *MAP2K4* (Supplementary information, Table S1). We examined drug sensitivity of the breast cancer cell line panel in relation to their genotypes. Given the frequent mutations in the MAPK pathway in breast cancer patients, we focused initially on drugs that act on this pathway. The drugs that are most advanced clinically are the MEK inhibitors, as exemplified by trametinib and selumetinib.^7,8^ Inhibition of MEK kinases in cancer cells has been shown to trigger complex feedback loops and pathway cross talk that can modulate drug responses (reviewed in ref. ^25^). The time frames in which these processes are activated are variable, but can take up to 72 h to become fully activated following MEK inhibition.^26^ We therefore used long-term cell proliferation assays to avoid that the early effects of MEK inhibition that take place when cells adjust to a new equilibrium confound the results. Such long-term cell proliferation assays may also resemble more closely the continuous exposure to drug that happens in vivo. Figure 1a shows that only two cell lines in the panel were sensitive to selumetinib (AZD6244): the *MAP2K4* mutant cell lines MDA-MB-134VI and MPE600 (colored red). To further study a possible relationship between *MAP2K4* mutations and responsiveness to MEK inhibition, we searched for additional *MAP2K4* mutant cell lines in the well-annotated Sanger Center cell line panel.^27^ We identified an additional 6 cancer cell lines of different organ types (large intestine, ovary, endometrium, pancreas) with homozygous mutations in *MAP2K4* (Supplementary information, Table S1). All were found to be sensitive to selumetinib, whereas six wild type control cell lines were resistant. (Fig. 1b). We also quantified cell proliferation using an Incucyte system that detects cell confluence over time. These data again indicate that selumetinib treatment reduces cell proliferation in *MAP2K4* mutant cells, but not in the wild-type cells (Fig. 1c). The *MAP2K4* mutant breast cancer cells were also sensitive to the MEK inhibitor trametinib and the ERK inhibitor SCH772984 (Fig. 1d, e).

**Fig. 2:**
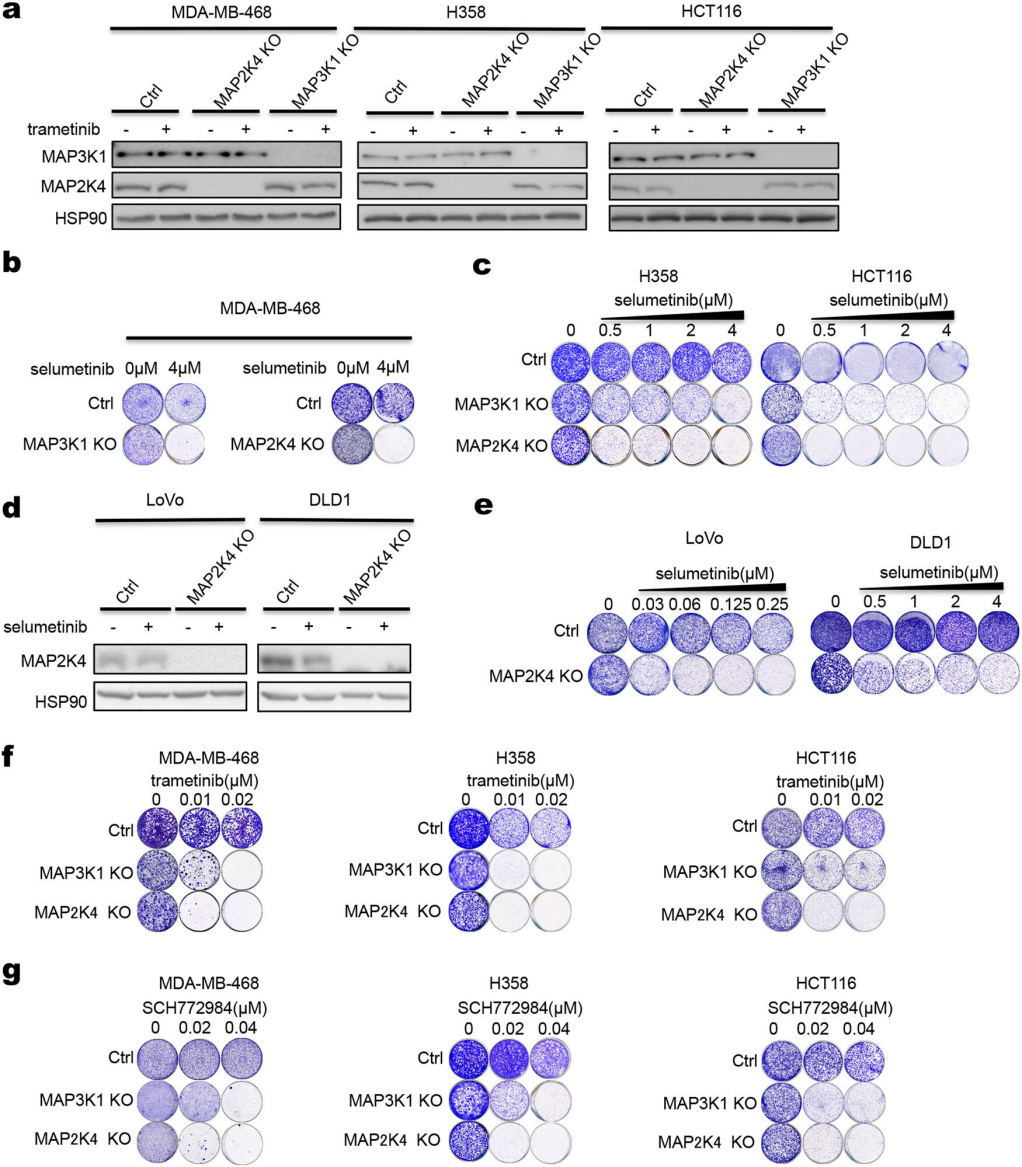
*MAP3K1* and *MAP2K4* knockout confer sensitivity to selumetinib. **a** *MAP3K1* or *MAP2K4* knockout cells were generated using a lentiviral CRISPR/Cas9 vector. Control and *MAP3K1* or *MAP2K4* knockout MDA-MB-468, H358 or HCT116 cells were treated with 0.02 μM trametinib for 72 h, and lysates were western blotted for MAP3K1 and MAP2K4. HSP90 served as a control. **b**, **c** Control and *MAP3K1* or *MAP2K4* knockout MDA-MB-468, H358 or HCT116 cells were cultured for 2 weeks in medium containing the indicated concentration of selumetinib. Then cells were fixed and stained. **d** Control and MAP2K4 knockout LoVo or DLD1 cells were treated with 2 μM selumetinib, lysates were western blotted for MAP2K4. HSP90 served as a control. **e** Control and MAP2K4 knockout LoVo or DLD1 cells were cultured for two weeks in medium containing the indicated concentration of selumetinib. Then cells were fixed and stained. **f**, **g** Control and *MAP3K1* or *MAP2K4* knockout MDA-MB-468, H358 or HCT116 cells were cultured for two weeks in medium containing the indicated concentration of trametinib (**f**) or SCH772984 (**g**). Then cells were fixed and stained

To ask whether *MAP2K4* loss-of-function mutations cause sensitivity to MEK inhibition, we generated a panel of isogenic knockout cells using CRISPR/Cas9 technology. MAP3K1 acts upstream of MAP2K4 in signaling and in the METABRIC breast cancer study, loss-of-function mutations in these genes is mutually exclusive (*p* < 0.001) (Supplementary information, Fig. S1a). We therefore included *MAP3K1* knockout in these analyses as well. MDA-MB-468 breast cancer, H358 lung cancer and HCT116 colon cancer cells in which *MAP3K1* or *MAP2K4* was knocked out all show a marked increase in sensitivity to selumetinib (Fig. 2a–c). Similar results were found for *MAP2K4* knockout LoVo and DLD1 colon cancer cells (Fig. 2d, e). *MAP3K1* or *MAP2K4* knockout also sensitize H358 cells to siRNA-mediated MEK1/2 knockdown (Supplementary information, Fig. S2a, b). Increased sensitivity of *MAP3K1*/*MAP2K4* knockout cells was also seen with the MEK inhibitors trametinib, binimetinib and the ERK inhibitor SCH772984 (Fig. 2f, g and Supplementary information, Fig. S2c). Re-expression of *MAP2K4* in *MAP2K4* mutant MPE600 breast cancer cells was not compatible with proliferation, consistent with the tumor suppressor nature of this gene (Supplementary information, Fig. S1b, c).

### Short-term versus long-term responses to MEK inhibitors

**Fig. 3:**
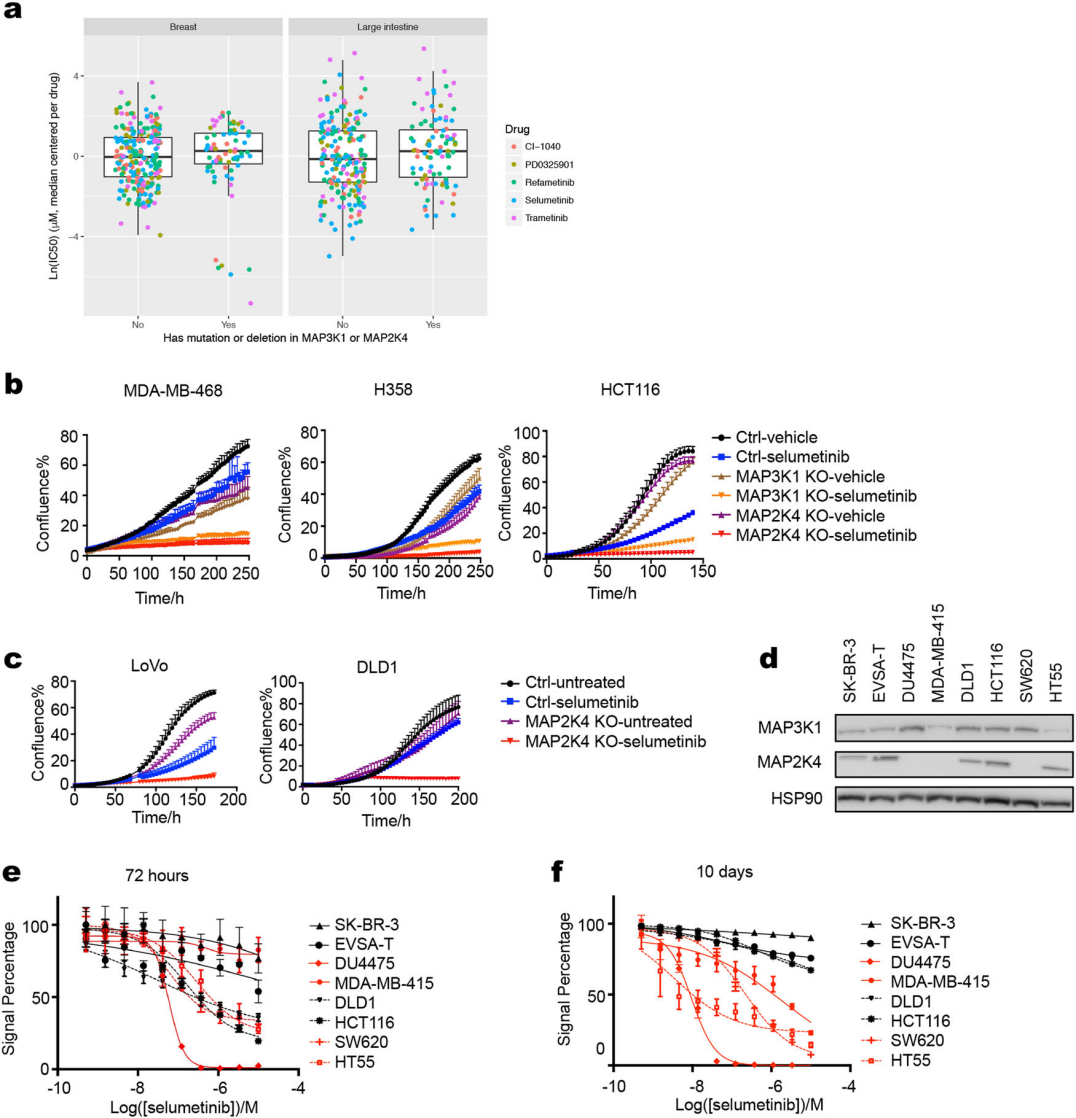
Short-term versus long-term responses to MEK inhibitors. **a** IC50 value of 5 different MEK inhibitors in breast (left) and colon cancer (right) cell line panel of Sanger drug screen data. **b, c** Cell proliferation curves of control and *MAP3K1* or *MAP2K4* knockout MDA-MB-468, H358, HCT116, LoVo and DLD1 cells were cultured in normal medium or medium containing 4 μM selumetinib. Percent confluence over time was monitored using IncuCyte. **d** Lysates of *MAP3K1* or *MAP2K4* mutant and wild-type cell lines were western blotted for MAP2K4 and MAP3K1. HSP90 served as a control. **e, f** The relationships between cell viability and response to a series of concentrations of selumetinib were examined for *MAP3K1* or *MAP2K4* mutant (colored red) and wild-type (colored black) breast and colon cancer cell lines after 72 h (**e**) or 10 days (**f**) of drug treatment

Most drug response assays on large cancer cell panels are performed over a 72-h time frame.^27,28^ As pointed out above, major cross talk and feedback mechanisms operate in this time frame, which may alter the outcome of short-term drug responses. Indeed, when we interrogated the Sanger cell line drug sensitivity data,^27^ we did not find a difference in sensitivity to five different MEK inhibitors as a function of their *MAP2K4* and *MAP3K1* mutation status (Fig. 3a). Note that in the time course experiment using four breast cancer cell lines, the MEK inhibitor sensitivity of the two *MAP2K4* mutant breast cancer cell lines was also not very apparent during the first 72 h of culture (Fig. 1c) and similar result was seen in the isogenic *MAP3K1* and *MAP2K4* knockout cells (Fig. 3b, c). To study the effects of short-term versus long-term drug exposure further, we determined the IC50 values of the MEK inhibitor selumetinib in a cell line panel including four *MAP2K4* or *MAP3K1 mutant* and four wild-type cell lines (Fig. 3d) using both ten-day and three-day assays (Supplementary information, Table S2). Figure 3f shows that in the 10-day assay, *MAP2K4* or *MAP3K1 mutant* cell lines tested were relatively sensitive to MEK inhibition (in red) compared to wild-type cell lines (in black). When the same cell lines were tested for selumetinib sensitivity in a 72-h assay, this difference in drug sensitivity was not evident (Fig. 3e). These divergent results in responsiveness to MEK inhibitor treatment beg the question whether the responses of cancer cells to MEK inhibitors in vivo resemble more the short-term or the long-term in vitro cell line responses. This question is addressed below.

### MEK inhibition activates a JNK-JUN feedback loop only in MAP3K1; MAP2K4 wild-type cells

**Fig. 4:**
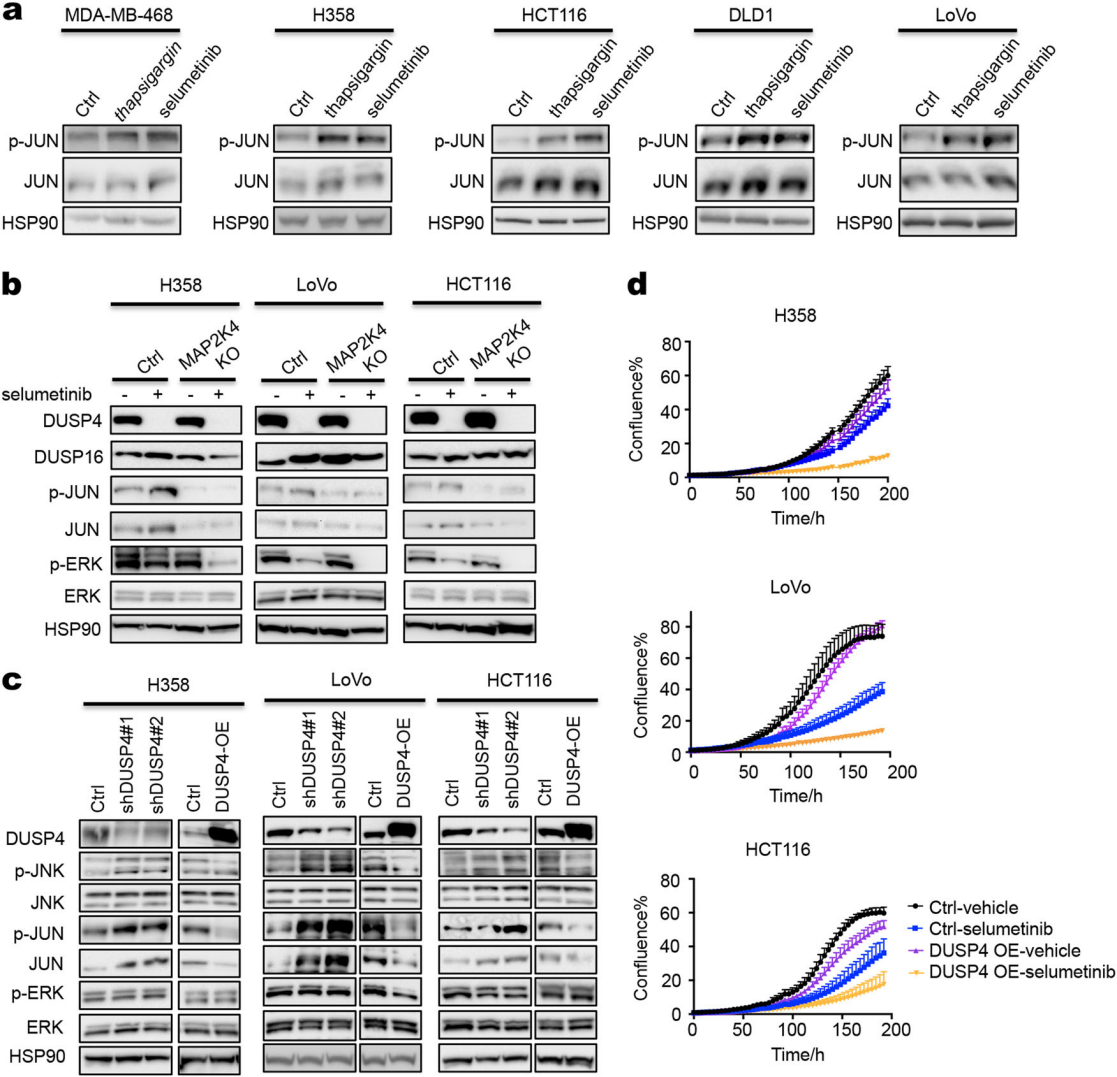
MEK inhibitor activates JNK-JUN signaling through suppression of DUSP4. **a** Five *MAP3K1/MAP2K4* wild-type cell lines were treated with 100 nM thapsigargin for 1 h or 2 μM selumetinib for 6 h, the levels of p-JUN and JUN were determined by western blot analysis. HSP90 served as a loading control. **b** Control and *MAP2K4* knockout H358, LoVo or HCT116 cells were treated with 2 μM selumetinib for 72 h and lysates were western blotted for DUSP4, DUSP16, p-JUN, JUN, p-ERK, ERK. HSP90 served as a control. **c** Two individual shRNAs targeting DUSP4 or a DUSP4 expression vector (DUSP4 OE) were introduced into H358, LoVo or HCT116 cells by lentiviral transduction. Lysates of control and *DUSP4* knockdown or overexpression H358, LoVo or HCT116 cells were western blotted for DUSP4, p-JNK, JNK, p-JUN, JUN, p-ERK, ERK. HSP90 served as a control. **d** Control and *DUSP4* overexpression H358, LoVo or HCT116 cells were cultured with normal medium or medium containing 0.25 μM (LoVo) or 4 μM (H358 and HCT116) selumetinib. Percent confluence over time was monitored using IncuCyte

**Fig. 5:**
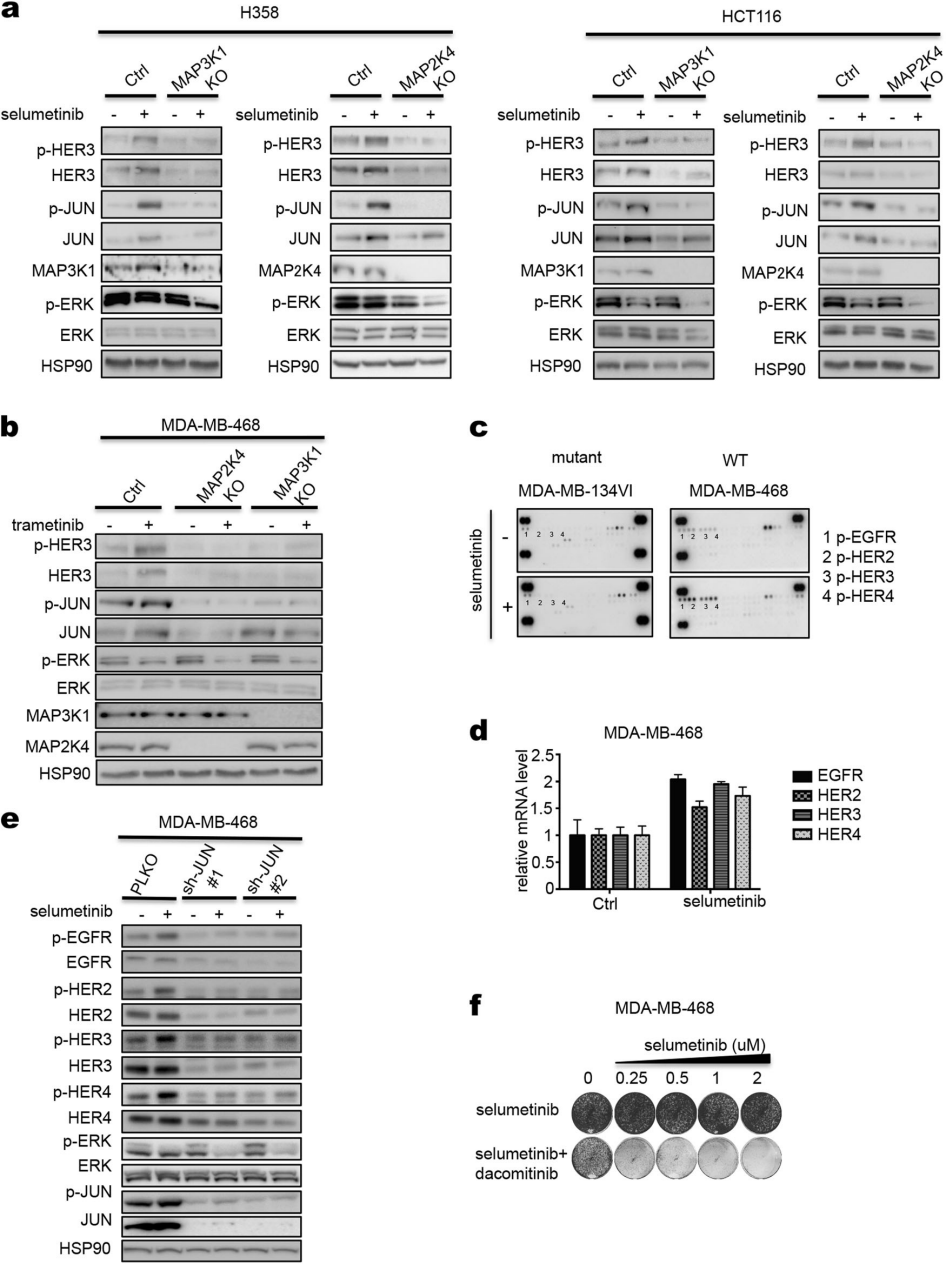
HER receptors are activated by MEK inhibitor in *MAP3K1; MAP2K4* wild-type cells. **a** Control and *MAP3K1* or *MAP2K4* knockout H358 or HCT116 cells were treated with 2 μM selumetinib for 72 h and lysates were western blotted for p-HER3, HER3, p-JUN, JUN, MAP2K4 or MAP3K1, p-ERK, ERK. HSP90 served as a control. **b** Control and MAP3K1 or MAP2K4 knockout MDA-MB-468 cells were treated with 0.02 μM trametinib for 72 h. The levels of p-HER3, HER3, p-JUN, JUN, p-ERK, ERK, MAP3K1 and MAP2K4 were determined by western blot analysis. **c** Two breast cancer cell lines of indicated MAP2K4 mutation status were treated with 2 μM selumetinib for 72 h and lysates were subjected to phospho-RTK activation analysis. Dots labeled 1-4 represent duplicate blots of p-EGFR, p-HER2, p-HER3 and p-HER4, respectively. **d** MDA-MB-468 cells were treated with selumetinib for 72 h, then RNA was extracted and qRT-PCR analysis was performed for HER receptor transcripts. **e** Two individual shRNAs targeting JUN were introduced into MDA-MB-468 cells by lentiviral transduction. Ctrl and JUN knockdown cells were treated with 2 μM selumetinib for 72 h. The levels of phospho-HER1-4 and HER1-4 receptors, p-JUN, JUN, p-ERK and ERK were determined by western blot analysis. HSP90 served as a loading control. **f** MDA-MB-468 cells were cultured for two weeks in medium containing increasing concentration of selumetinib alone, dacomitinib (8 nM) alone, or combination of selumetinib and dacomitinib. After this, cells were fixed and stained

The MAP3K1 and MAP2K4 kinases activate the JUN transcription factor through the JNK kinases.^24^ JUN in turn activates several RTKs, including EGFR,^29–32^ HER2^33^ and PDGFRB.^34^ To study the mechanism of sensitivity of *MAP2K4* mutant cancer cells to MEK inhibition, we analyzed ERK and JNK signaling in *MAP2K4* mutant and wild-type cells. Consistent with the data of others,^22,23^ selumetinib treatment resulted in activation of JNK kinase in 5 different cell lines to the same extent as thapsigargin, as evidenced by an increase in phosphorylation of its downstream target JUN (p-JUN) (Fig. 4a). Activation of JUN by selumetinib is dependent on MAP3K1-MAP2K4 signaling, as H358, LoVo and HCT116 cells having knockout of *MAP3K1* or *MAP2K4* fail to activate JUN after MEK inhibition (Figs. 4b, 5a). Similar results were seen in MDA-MB-468 breast cancer cells (Fig. 5b). Conversely, expression of *MAP2K4* in *MAP2K4* mutant MPE600 cells, although incompatible with proliferation (Supplementary information, Fig. S1c), did result in re-activation of JUN (Supplementary information, Fig. S1b). Consistent with published data,^22,23,35^ inhibition of MEK caused a rapid disappearance of DUSP4, both in *MAP2K4* wild type and knockout cells (Fig. 4b). In agreement with the established role for DUSP4 in JNK regulation, knockdown of *DUSP4* caused an increase in JNK-JUN signaling (Fig. 4c) and conversely, ectopic expression of *DUSP4* inhibited JNK-JUN signaling and consequently increased sensitivity to selumetinib in 3 different cell line models (Fig. 4c, d).

Activation of HER3 is responsible for the intrinsic resistance of lung and colon cancer cells to MEK inhibitors.^26^ Indeed, treatment of H358 and HCT116 cells with selumetinib resulted in induction of the active, phosphorylated form of HER3 (p-HER3), whose downstream signaling precludes efficient suppression of signaling to the ERK kinases downstream of MEK^26^ (Fig. 5a). Importantly, activation of HER3 was attenuated by *MAP3K1* or *MAP2K4* knockout and resulted in downregulation of p-ERK, indicating efficient inhibition of MEK kinase activity (Fig. 5a). Similar results were seen in MDA-MB-468 breast cancer cells (Fig. 5b). HER3 forms active heterodimeric complexes with EGFR, HER2 and HER4. Indeed, an unbiased survey of the RTKs that become activated in response to MEK inhibition shows that MDA-MB-468 cells activate all four HER receptors, also at the level of transcription, but this activation is not seen in *MAP2K4* mutant MDA-MB-134VI cells (Fig. 5c, d). The role of JUN in the activation of the HER RTKs is evident from the finding that knockdown of JUN with two different shRNA vectors resulted in suppression of the MEK inhibitors-induced activation of all HER receptors and consequently also in more efficient p-ERK inhibition (Fig. 5e). That MDA-MB-468 cells are insensitive to MEK inhibition due to activation of HER RTKs is also supported by the notion that MDA-MB-468 cells are not growth-inhibited by MEK inhibition alone, but do respond to co-treatment with MEK inhibitor and a small molecule pan-HER inhibitor (dacomitinib) (Fig. 5f). The role of JNK-JUN signaling in resistance to MEK inhibitors is further supported by the finding that knockdown of JUN with shRNAs confers sensitivity to MEK inhibition and the notion that two different JNK kinase inhibitors synergize with MEK inhibition in breast and colon cancer cells (Supplementary information, Fig. S3a–c).

### *MAP3K1* and *MAP2K4* mutations confer sensitivity to MEK inhibitor in vivo

**Fig. 6:**
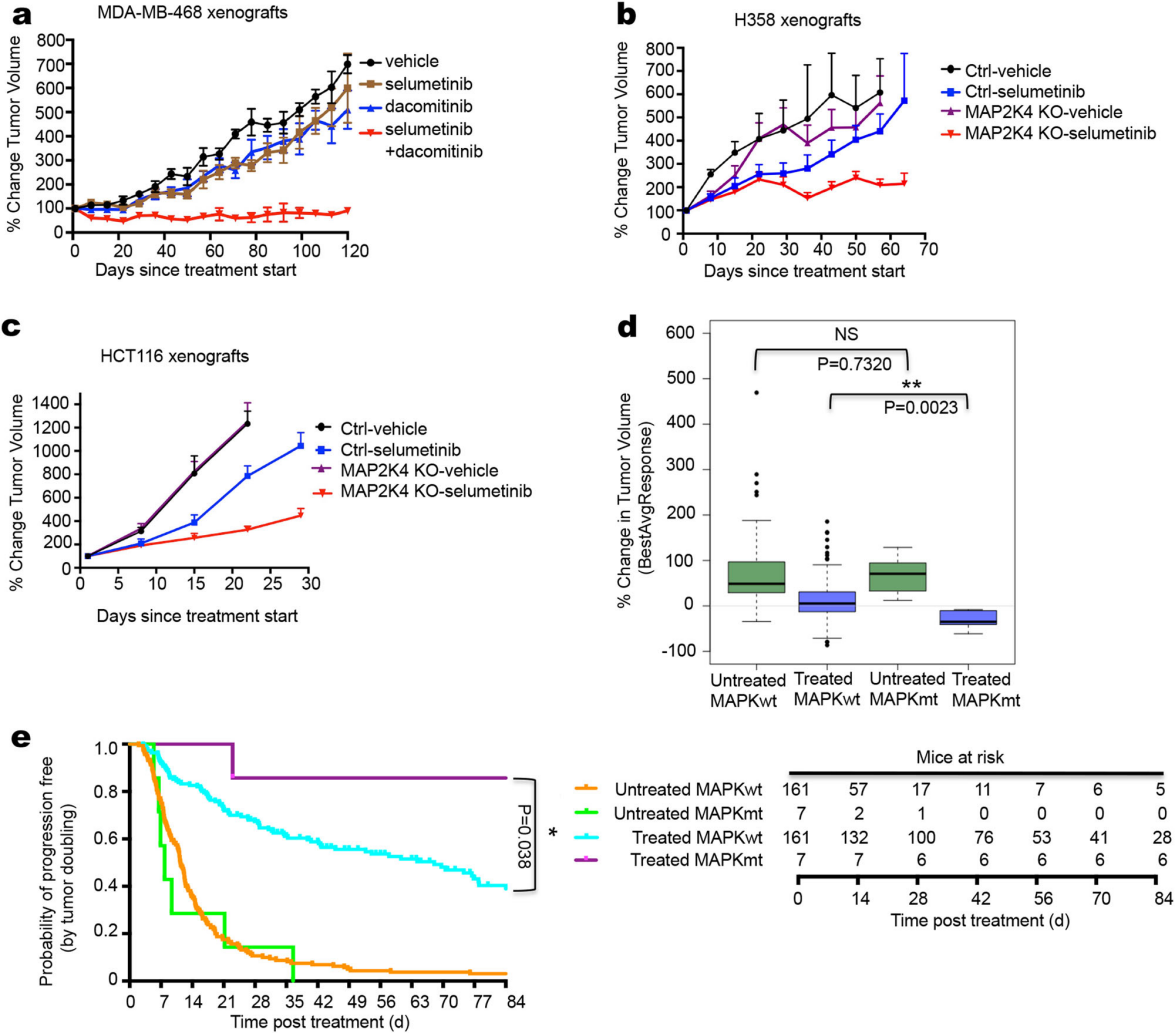
*MAP3K1* and *MAP2K4* mutant confer sensitivity to MEK inhibitor in vivo. **a** MDA-MB-468 cells were injected into nude mice. Once tumors reached 100 mm^3^, mice (six per group) were treated with vehicle, selumetinib (20 mg/kg/day), dacomitinib (3.75 mg/kg/day), or both drugs in combination (selumetinib 20 mg/kg/day + dacomitinib 3.75 mg/kg/day). The mean percentage change from the initial tumor volume is shown. Error bars represent standard error of the mean (SEM). **b, c** H358 **b** and HCT116 **c** cells, both Ctrl and *MAP2K4* knockout cells, were injected into nude mice. Once tumors reach 100 mm^3^, mice (six per group) were treated with vehicle or selumetinib (20 mg/kg/day). The mean percentage change from the initial tumor volume is shown. Error bars represent standard error of the mean (SEM). **d** PDX drug response and mutation data were obtained from ref. ^36^. Mutation data and binimetinib response data were available for 168 PDX models. Shown in the blue box plots is best average binimetinib response in 7 PDX tumors having either a *MAP3K1* or a *MAP2K4* mutation versus 161 without mutation in these genes; green box plots show tumor growth rate without drug. **e** Kaplan-Meier PFS curve of wild-type (*n* = 161) and *MAP3K1* or *MAP2K4* mutant (*n* = 7), untreated and binimetinib treated among the 168 PDX models. Numbers of animals at risk over time in each group is also shown

To test our findings in vivo, we injected *MAP3K1/MAP2K4* wild type MDA-MB-468 cells into nude mice. When tumors reached a volume of 100 mm^3^, drug treatment was started. Figure 6a shows that these cells failed to respond to treatment with selumetinib, but did respond to the combination of selumetinib and dacomitinib, consistent with the in vitro data. Xenografted H358 and HCT116 tumors also failed to respond to selumetinib, but became responsive to the drug when *MAP2K4* was knocked out (Fig. 6b, c). A collection of 1075 xenografted patient-derived tumors (PDX) was recently generated and the responses of these PDX tumors to 36 drugs were documented in relation to the mutations carried by these tumors.^36^ We identified in this cohort 7 PDX tumors with mutation in *MAP3K1* or *MAP2K4* and 161 tumors that were wild type for both genes and for which responses to the MEK inhibitor binimetinib are documented (Supplementary information, Table S3). Consistent with our in vitro and in vivo findings, PDX models having mutations in *MAP3K1* or *MAP2K4* were significantly more sensitive to binimetinib than their wild type counterparts, with only the mutant tumors showing a decrease in tumor volume over time (Fig. 6d, p = 0.0023). These same data are also represented in a Kaplan-Meier curve (Fig. 6e). Analyzed in this way, these data again highlight that the MAPK mutant tumors have the same growth rate as their wild type counterparts, but differ only in response to binimetinib treatment (p = 0.038). The individual responses of the seven PDX models to binimetinib are shown in Supplementary information, Fig. S4a. We also used breast cancer patient-derived tumor xenograft models for which both genomic information and drug responses are documented.^37^ Since there was a paucity of models with mutations in *MAP3K1* or *MAP2K4* in this collection, we selected four models having varying degrees of *MAP3K1* and/or *MAP2K4* copy number loss (Supplementary information, Table S4). In vivo, three models were sensitive to selumetinib (VHIO0098, HCI009 and STG316), whereas one was relatively resistant (STG139) (Supplementary information, Fig. S4b). We note that copy number loss is not a guarantee for complete loss-of-function of the gene, which may explain the resistance in the STG139 model. Given the overall poor responses of PDX tumors to selumetinib,^38^ these data further support our notion that loss of *MAP3K1* or *MAP2K4* confers sensitivity to MEK inhibitors.

## Discussion

**Fig. 7:**
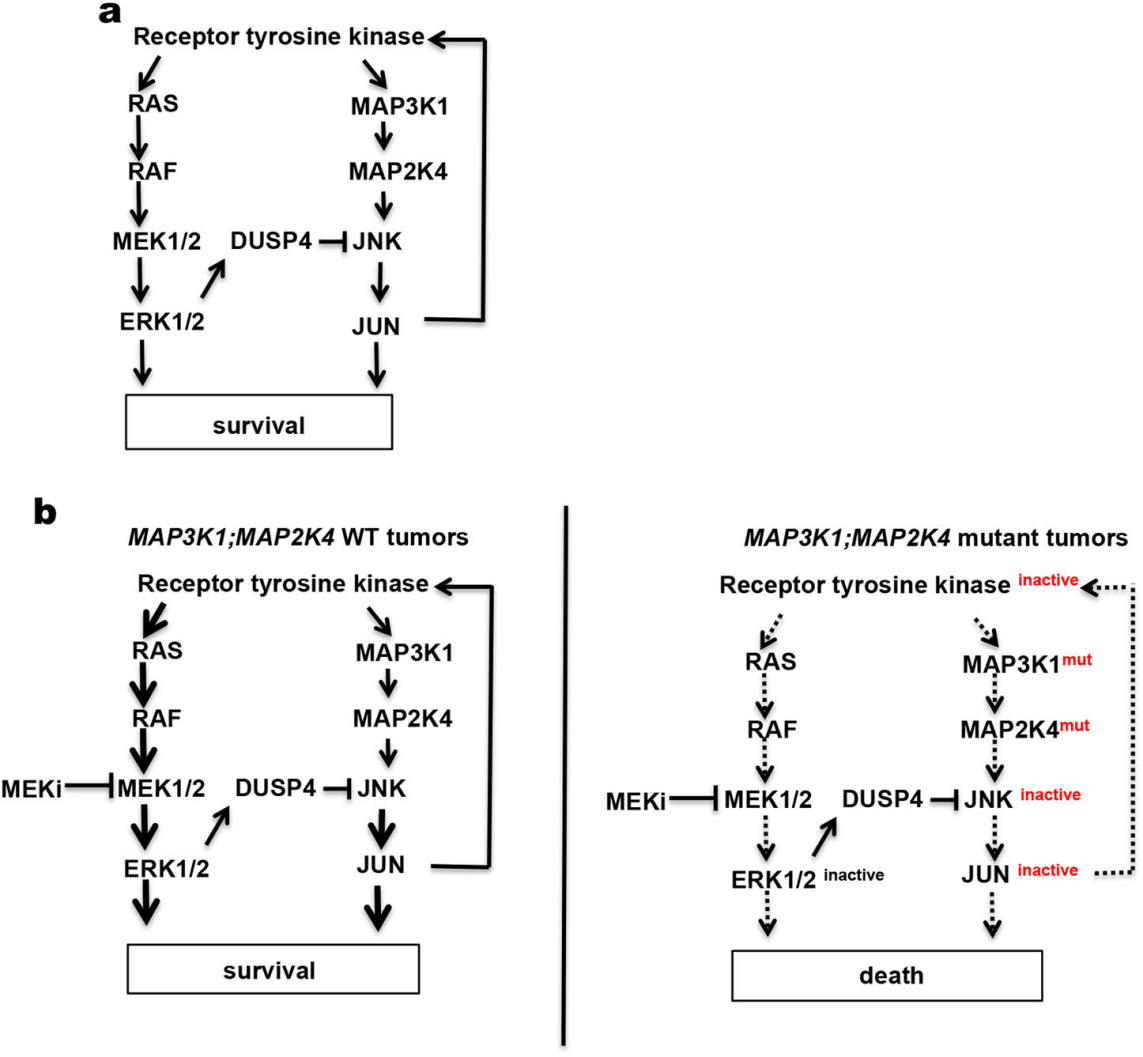
Model of MAPK signaling pathway in *MAP3K1; MAP2K4* wild-type or mutant cells. **a** Schematic representation of cross-talk of the MEK-ERK and JNK signaling pathways. **b** MEK-ERK and JNK signaling pathways in MAP3K1;MAP2K4 wild-type (left) or mutant (right) tumors with MEK inhibitor treatment

Most clinical studies with MEK inhibitors have yielded disappointing results, due at least in part to the paucity of biomarkers of MEK inhibitor sensitivity. We report here that inhibition of MEK kinases in both *RAS* wild type and *RAS* mutant tumors results in a feedback activation of the parallel MAP3K1-MAP2K4-JNK-JUN pathway. This in turn leads to activation of a number of HER family receptor tyrosine kinases whose downstream signaling limits the efficacy of MEK inhibitor monotherapy (Fig. 7a).^26^ It was shown recently that tumors that lack a wild type *KRAS* allele have increased MEK inhibitor sensitivity.^39^ We show here that cancer cells that have inactivating mutations in *MAP3K1* or *MAP2K4* are sensitive to MEK inhibitor monotherapy by disabling the positive feedback loop that limits drug responsiveness (Fig. 7b). Such feedback loops are frequent in cancer, as the efficacy of BRAF inhibitors in *BRAF* mutant colon cancer is also blunted by an EGFR-dependent feedback mechanism.^40,41^ A study in *BRAF* mutant colon cancer patients indicates that inhibition of this feedback loop dramatically increases clinical response to BRAF inhibitors.^42^ Moreover, in *KRAS* mutant tumors, inhibition of HER activation following MEK inhibition, which we show here requires functional MAP3K1 and MAP2K4, enhances responses to MEK inhibitors (Fig. 7b).^26^ It therefore seems likely that tumors in which this MEK inhibitor feedback loop is disabled by mutation in either *MAP3K1* or *MAP2K4* will be intrinsically sensitive to MEK inhibition. Such mutations are present in some 100,000 patients diagnosed in the US alone annually^2^ (http://www.cbioportal.org). As such, our data provide a DNA-guided biomarker strategy to identify patients that are most likely to respond to MEK inhibition. Our data also predict that inhibitors of the MAP3K1, MAP2K4 or JNK kinases should also show synergy with MEK inhibition in a variety of cancers. Perhaps more strikingly, our data also predict that inhibition of MAP3K1 or MAP2K4 with small molecules (which do not exist currently) would be highly synergistic with MEK inhibition. This appears counter-intuitive as MAP3K1 and MAP2K4 are encoded by genes having tumor suppressor-like properties.

## Materials and methods

### Cell lines and cell culture, inhibitors and antibodies

Breast cancer cell lines EVSA-T, HCC1187, MDA-MB-134VI, MDA-MB-468, MPE600 and SK-BR-3 were a kind gift of Dr. Mieke Schutte (Josephine Nefkens Institute, Erasmus University Medical Center, Rotterdam, The Netherlands). H358, HCT116, LoVo, DLD1, SW620, SW403, OAW-28, KLE, AsPC-1, BxPC-3, SKOV3, OVCAR3, MIAPaCa-2 and YAPC cell lines were purchased from American Type Culture Collection (ATCC). All the cell lines were cultured in RPMI 1640 medium supplemented with 10% fetal bovine serum, glutamine and Penicillin (Gibco) at 37 °C in 5% CO_2_ and authenticated through STR profiling.

Selumetinib (S1008), trametinib (S2673), SCH772984 (S7101), JNK-IN-8 (S4901) and dacomitinib (S2727) were purchased from Selleck Chemicals.

Antibodies against p-JNK (T183/Y185) (4668), JNK (9252), p-JUN (S63) (2361), JUN (2315), MAP2K4 (9152), DUSP4 (5149), DUSP16 (5523), p-ERBB2 (Y1221/1222) (2243), ERBB2 (4290), p-ERBB3 (Y1222) (4784), ERBB3 (4754), p-ERBB4 (Y1284) (4757) and ERBB4 (4795) were purchased from Cell Signaling Technology. Antibodies against p-ERK (E-4), ERK1 (C-16), ERK2 (C-14) and HSP90 (H-114) were purchased from Santa Cruz Biotechnology. Antibodies against p-EGFR (Y1068) (ab5644) and MAP3K1 (ab55653) were purchased from Abcam. Antibody against EGFR (06-847) was purchased from Millipore.

### Growth inhibition assays

Cell lines were cultured and seeded into 384-well plates (1000–3000 cells per well) or 96-well plates (500–1000 cells per well). After 24 h incubation, three-fold serial dilutions of drugs were added to final drug concentrations ranging from 0.0005–10 μM. Cell viability was measured with the CellTiter-Blue assay (Roche) after treatment with drug for 72 h or 10 days (medium was changed after 3 days). The relative survival of different cell lines in the presence of drug was normalized against control conditions (untreated cells) after subtraction of background signal.

### Cell proliferation assays

Cells were cultured and seeded into 6-well plates at density of 1–2 × 10^4^ cells per well, depending on growth rate and were cultured in the medium containing the indicated drugs for 2 weeks (medium was changed twice a week). After this, cells were fixed with 4% formaldehyde in PBS and stained with 0.1% crystal violet in water.

Incucyte proliferation assays were carried out in 96-well plates at a density of 500–1000 cells per well. 24 h later, drugs were added using HP D300 Digital Dispenser (HP) at indicated concentrations. Cells were imaged every 4 h in IncuCyte ZOOM (Essen Bioscience). Phase-contrast images were collected and analyzed to detect cell proliferation based on cell confluence.

### Protein lysate preparation and western blot

Cells were plated in complete medium. After 24 h incubation, cells were treated under indicated conditions. Then the cells were washed twice with PBS and lysed in RIPA buffer supplemented with Complete Protease Inhibitors (Roche), Phosphatase Inhibitor Cocktails II and III (Sigma). The lysates were then resolved by electrophoresis in Novex NuPAGE gels and followed by western blotting.

### siRNA- and shRNA-mediated gene knockdown

siRNAs targeting MEK1 and MEK2 from Human siGenome SMARTpool library (Dharmacon) were used in siRNA-mediated gene silencing. H358 cells were transfected using DharmaFECT transfection reagent #1 and 25 nM siRNA. The lentiviral-based RNAi Consortium (TRC) human genome-wide shRNA collection (TRCHs1.0) was used in making gene knockdown cell lines. Individual lentiviral plasmids containing shRNAs against JUN or DUSP4 were collected from TRC library. The CCSB-Broad lentiviral collection of human ORFs was used in making gene overexpression cell lines. The lentiviruses were produced as described at http://www.broadinstitute.org/rnai/public/resources/protocols. In brief, HEK293T cells were transfected with lentiviral vectors using calcium phosphate method. Lentiviral supernatants were collected and transduced into target cells with polybrene (1 mg/mL). Stable gene knockdown or overexpression cell lines were selected with puromycin (2 μg/mL) or Blasticidin (10 μg/mL).

### CRISPR/Cas9-mediated gene knockout

The lentiviral-based CRISPR/Cas9-mediated gene knockout cell lines were produced as described at http://genome-engineering.org/gecko. In brief, sequence of individual sgRNAs against *MAP3K1* and *MAP2K4* were collected from genome-scale CRISPR knock-out (GeCKO) libraries, and then cloned to LentiCRISPRv2 vector. To make lentivirus, HEK293T cells were co-transfected by lentiCRISPRv2 plasmids contacting individual sgRNAs and packaging plasmids. Lentiviruses were collected and transduced into target cells with polybrene (1 mg/mL). After puromycin (2 μg/mL) selection, single clones were cultured and knockout clones were identified.

### RNA isolation and analysis

Cells were harvested and total RNA was isolated using Trizol (Invitrogen). For real-time PCR analysis, cDNA was synthesized from total RNA using Maxima Universal First Strand cDNA Synthesis Kit (Thermo scientific). The resulting cDNA was subjected to PCR analysis with gene-specific primers using Biosystems 7500 Real-Time PCR Systems (life technologies). The housekeeping gene GAPDH was used as the internal control. The PCR products were detected by measurement of the SYBR Green (Roche). The primer sequences are as follow: EGFR_forward: TCCTCTGGAGGCTGAGAAAA; EGFR_reverse: GGGCTCTGGAGGAAAAGAAA; HER2_forward: AGCATGTCCAGGTGGGTCT; HER2_reverse: CTCCTCCTCGCCCTCTTG; HER3_forward, GGGGAGTCTTGCCAGGAG; HER3_ reverse: CATTGGGTGTAGAGAGACTGGAC; HER4_forward: GCCTCTGGAGAATTTACGCAT; HER4_reverse: GGGTTCCGAACAATATCTTGCC; GAPDH_forward: AAGGTGAAGGTCGGAGTCAA; GAPDH_reverse: AATGAAGGGGTCATTGATGG.

### phospho-RTK activation analysis

The phospho-RTK activation analysis was done following the manufacturers’ instruction of Human Phospho-Receptor Tyrosine Kinase Array Kit (R&D). Briefly, cells were lysed and incubated with blocked array membranes overnight. Then the array membranes were washed and incubated with Anti-Phospho-Tyrosine-HRP Detection Antibody. The arrays were then washed and processed using a luminol based chemical reagent, and followed by X-ray film exposure.

### In vivo mouse xenograft and PDX studies

Dacomitinib and selumetinib were dissolved in cremophor EL/DMSO (Sigma). All animals were performed according to protocols approved by the Animal Ethics Committee of the Netherlands Cancer Institute in accordance with the Dutch Act on Animal Experimentation. MDA-MB-468 cells (3.5 × 10^6^ cells per mouse) were injected subcutaneously in the right posterior flank of 7-week-old immunodeficient Balb/C female nude mice. Tumor formation was monitored twice a week. When the tumor volume reached approximately 100 mm^3^, mice were randomised (six mice per group) either treated orally 5 days on and 2 days off with vehicle, selumetinib (20 mg/kg of body weight by daily gavage), dacomitinib (3.75 mg/kg of body weight by daily gavage) or their combination at the same dose as monotherapy. H358 Ctrl and *MAP2K4* knockout cells (5 × 10^6^ cells per mouse) or HCT116 Ctrl and *MAP2K4* knockout cells (1 × 10^6^ cells per mouse) were injected subcutaneously in the right posterior flank of 7-week-old immunodeficient Balb/C female nude mice. Tumor formation was monitored twice a week. When the tumor volume reached approximately 100 mm^3^, mice were randomly (six mice per group) either treated orally with vehicle or selumetinib (20 mg/kg of body weight by daily gavage). The PDX model was generated as described before.^34^ Further information on the models can be found here: http://caldaslab.cruk.cam.ac.uk/bcape/. PDXs were randomly distributed into the two arms of the study (*n* = 3–6 mice per group) and treated as above with vehicle or selumetinib.

### Statistic analysis

All statistical tests were performed using the Wilcoxon test, using R 3.4.2 (https://www.R-project.org).

## Electronic supplementary material

Figure S1

Figure S2

Figure S3

Figure S4

Table S1

Table S2

Table S3

Table S4
