# Supplementary material for: MAP3K1 and MAP2K4 mutations are associated with sensitivity to MEK inhibitors in multiple cancer models
Source: Cell Res. 2018 May 24;28(7):719–29. doi: 10.1038/s41422-018-0044-4 (PMC6028652; doi:10.1038/s41422-018-0044-4)
Supplement: Supplementary file 1 — Figure S1 [file 41422_2018_44_MOESM1_ESM.pdf]

Figure S1

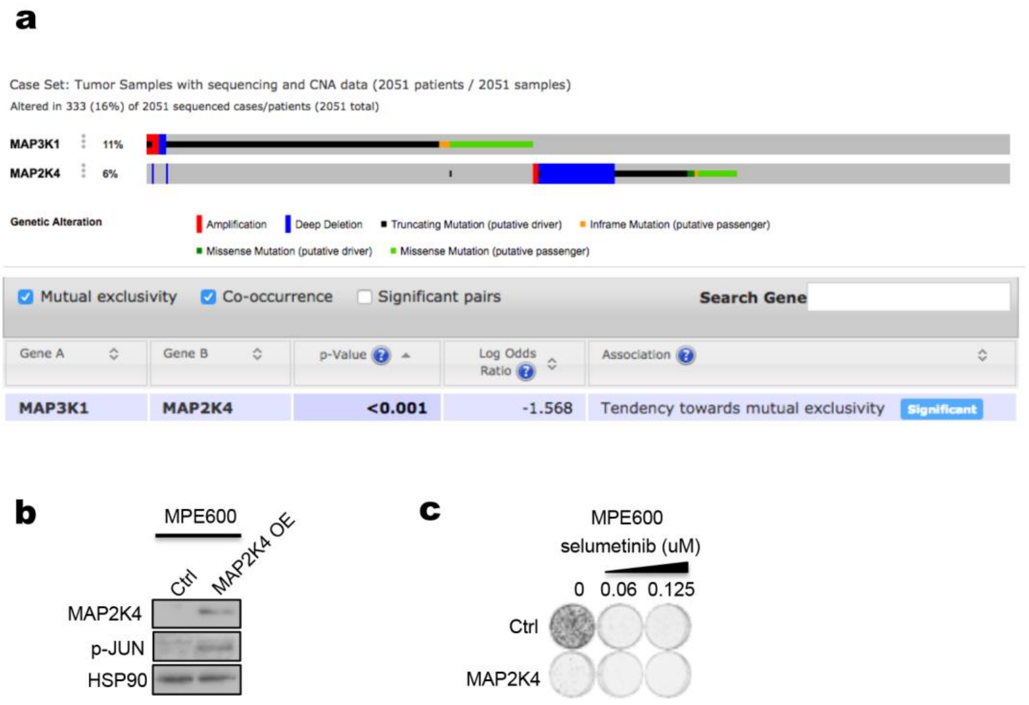

Figure S1. *MAP3K1* and *MAP2K4 mutation status.*

A. *MAP3K1* and *MAP2K4* mutation status and mutual exclusivity analysis in METABRIC breast cancer study obtained from http://www.cbioportal.org.

B. Lysates of control and MAP2K4 overexpression MPE600 cells were western blotted for p-JUN and MAP2K4. HSP90 served as a control.

C. Control and MAP2K4 overexpression MPE600 cells were cultured for two weeks in medium containing the indicated concentration of selumetinib. Then cells were fixed and stained.
