# Supplementary material for: MAP3K1 and MAP2K4 mutations are associated with sensitivity to MEK inhibitors in multiple cancer models
Source: Cell Res. 2018 May 24;28(7):719–29. doi: 10.1038/s41422-018-0044-4 (PMC6028652; doi:10.1038/s41422-018-0044-4)
Supplement: Supplementary file 2 — Figure S2 [file 41422_2018_44_MOESM2_ESM.pdf]

Figure S2

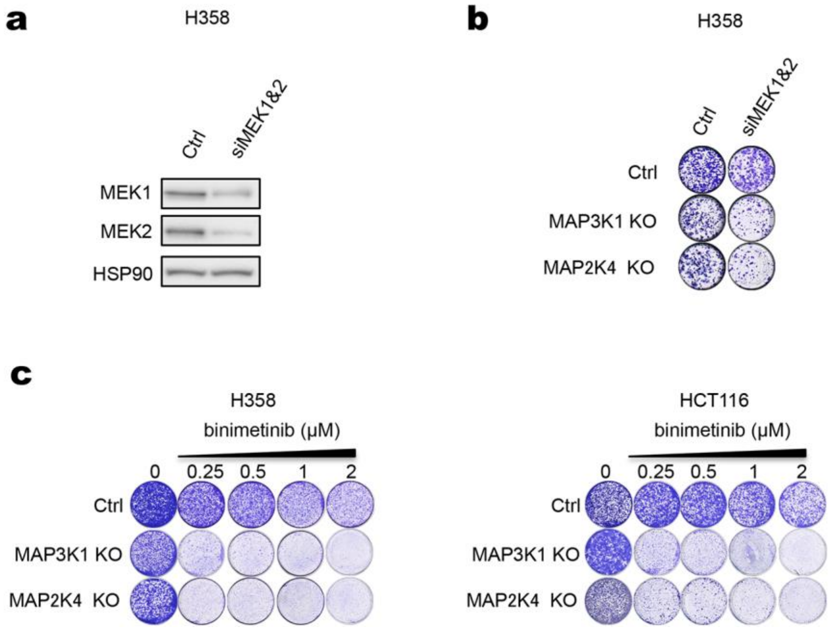

Figure S2. *MAP3K1* and *MAP2K4* knockout confer sensitivity to MEK inhibition.

A. H358 cells were co-transfected with siMEK1 and siMEK2, MEK1 and MEK2 knockdown was determined by Western blotting. HSP90 served as a control.

B. Control and *MAP3K1* or *MAP2K4* knockout H358 cells were transfected with scramble siRNA (control) or co-transfected with siMEK1 and siMEK2 for 7 days, then cells were fixed and stained.

C. Control and *MAP3K1* or *MAP2K4* knockout H358 and HCT116 cells were cultured for two weeks in medium containing the indicated concentration of binimetinib. Then cells were fixed and stained.
