# Supplementary material for: MAP3K1 and MAP2K4 mutations are associated with sensitivity to MEK inhibitors in multiple cancer models
Source: Cell Res. 2018 May 24;28(7):719–29. doi: 10.1038/s41422-018-0044-4 (PMC6028652; doi:10.1038/s41422-018-0044-4)
Supplement: Supplementary file 3 — Figure S3 [file 41422_2018_44_MOESM3_ESM.pdf]

Figure S3

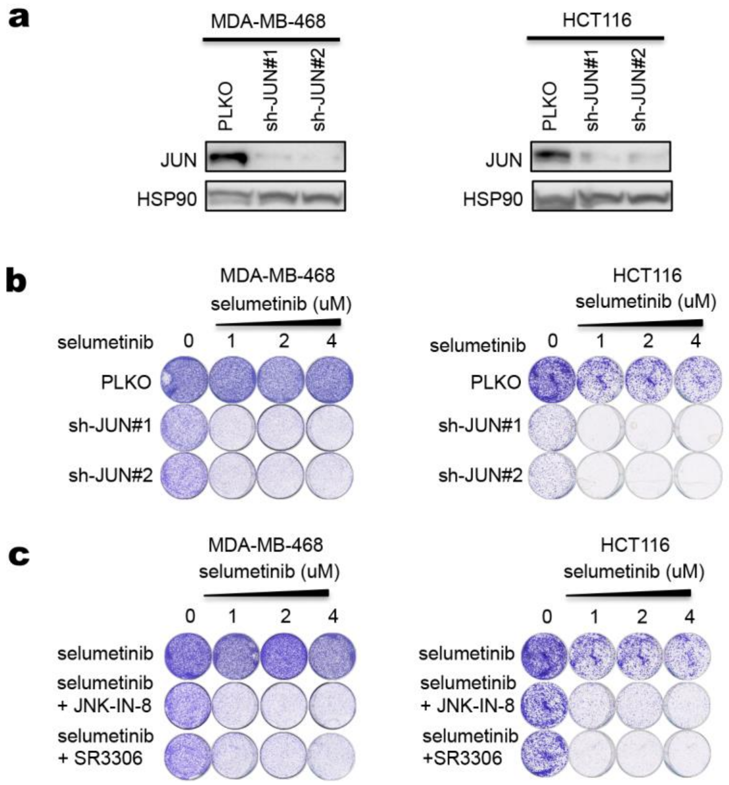

Figure S3. *JUN knockdown confer sensitivity to selumetinib.*

A. Two individual shRNAs targeting JUN were introduced into MDA-MB-468 and HCT116 cells by lentiviral transduction. JUN knockdown was determined by Western blotting. HSP90 served as a control.

B. Control and JUN knockdown MDA-MB-468 and HCT116 cells were cultured for two weeks in medium containing the indicated concentration of selumetinib. Then cells were fixed and stained.

C. MDA-MB-468 cells were pretreated with 0.25μM JNK-IN-8 or 0.25μM SR3306 and HCT116 cells were pretreated with 0.5μM JNK-IN-8 or 0.5μM SR3306 for one week. Then cells were seeded and cultured for two weeks in medium containing increasing concentration of selumetinib alone, JNK-IN-8 alone, SR3306 alone, or combination of selumetinib and JNK-IN- 8 or SR3306. After this, cells were fixed and stained.
