# Supplementary material for: MAP3K1 and MAP2K4 mutations are associated with sensitivity to MEK inhibitors in multiple cancer models
Source: Cell Res. 2018 May 24;28(7):719–29. doi: 10.1038/s41422-018-0044-4 (PMC6028652; doi:10.1038/s41422-018-0044-4)
Supplement: Supplementary file 4 — Figure S4 [file 41422_2018_44_MOESM4_ESM.pdf]

Figure S4

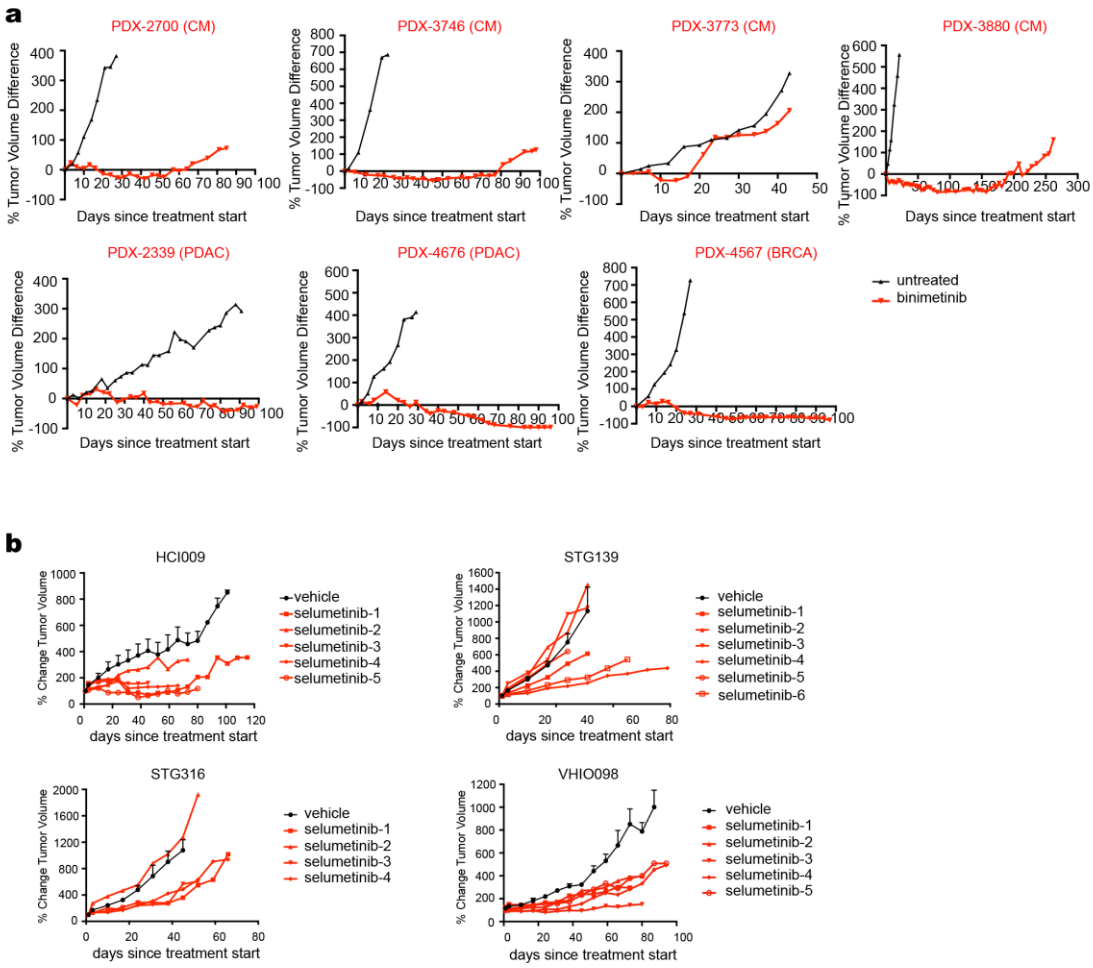

Figure S4. *MEK inhibitor responses of PDX mouse models*

A. Binimetinib response data of 7 *MAP3K1* or *MAP2K4* mutant PDX tumors were obtained from ref.[36]. The percentage change from the initial tumor volume of untreated (black) and binimetinib treated (red) is shown.

B. Indicated PDX mouse models were treated with vehicle or selumetinib (20mg/kg/day). For vehicle treated animals, the mean change from the initial tumor volume is shown. Error bars represent standard error of the mean (SEM). For drug treated animals, the percentage change is shown per individual animal.
